# Nonsteroidal antiinflammatory drug-exacerbated respiratory disease: molecular mechanism, management and treatment

**DOI:** 10.3389/falgy.2024.1462985

**Published:** 2024-11-27

**Authors:** J. J. Ley-Tomas, A. M. Xicotencatl-Tellez, M. L. García-Cruz, M. A. Jiménez-Chobillon

**Affiliations:** IAPA ’s Clinic, Department of Otorhinolaryngology–Head and Neck Surgery, Instituto Nacional de Enfermedades Respiratorias, Ismael Cosío Villegas, Ciudad de México, México

**Keywords:** aspirin exacerbated respiratory disease, AERD, N-ERD, Samter's triad, chronic rhinosinusitis, nasal polyposis

## Abstract

It has been estimated that Nonsteroidal Anti-inflammatory drug (NSAID) Exacerbated Respiratory Disease (N-ERD) previously named as Aspirin Exacerbated Respiratory Disease (A-ERD) affects around 1.4 million persons in the United States. Its prevalence in asthmatic patients has widely been underestimated, as a considerable number of patients would need an aspirin provocation test to confirm the diagnosis. N-ERD physiopathology is somehow complex, but basically involves an imbalance in the arachidonic acid metabolite pathway. The syndrome is characterized by the presence of asthma, chronic rhinosinusitis with nasal polyposis (CRSwNP) and NSAID and aspirin intolerance. Despite maximal and comprehensive medical treatment, the disease tends to be severe, with difficult to treat asthma and highly aggressive and recurrent ethmoidal polyposis. Recently, monoclonal antibodies aimed at reducing type 2 inflammation have demonstrated very promising results on disease control. The goal of this review is to provide the most recent published advances and evidence on physiopathology, diagnostic protocols and therapeutic strategies of N-ERD.

## 1 Introduction

Aspirin hypersensitivity reactions were first reported in medical literature more than a century ago (1), but it was not until 1922 that the classical triad of asthma, chronic rhinosinusitis with nasal polyposis (CRSwNP) and aspirin intolerance was first described by Widal et al. (2). More than forty years later, in 1968, Samter and Beer fully described the clinical syndrome characterized by CRSwNP, asthma and hypersensitivity to aspirin and cyclooxygenase-1 (COX-1) inhibitors (2, 3). This syndrome has also been named Aspirin Exacerbated Respiratory Disease (A-ERD), and more recently NSAID Exacerbated Respiratory Disease (N-ERD).

N-ERD physiopathology remains somewhat occult, an idiopathic dysfunction of arachidonic acid metabolism causes an imbalance in the synthesis of eicosanoids and prostaglandins (PG) that finally results in a non-allergic hypersensitivity reaction (4).

The objective of this review is to summarize the most recent scientific evidence on the physiopathology, natural history, diagnosis, and therapeutic options of N-ERD.

## 2 Epidemiology

Worldwide, there is very limited epidemiologic information, studies from the United States report that around 1.4 million people in that country suffer from N-ERD, a 1.9% in a European multicenter study, 1.2% in Finland and 1.3% in Sweden, and a limited number of studies population-based in Poland, Australia (5). Some of the reasons that could explain the absence of this type of studies are that the relationships between dyspnea, asthma and rhinitis induced by NSAIDs in N-ERD and the sub phenotypes of N-ERD are incomplete, in addition to the lack of knowledge of the presentation of symptoms, for example, patients with asthma and aspirin intolerance who do not present with sinonasal polyposis, during medical evaluation, the possibility of N-ERD is often ruled out without proper follow-up. The impact on public health lies in sub-optical medical and surgical treatments, substantially increasing spending on the care of these patients.

The disease affects around 9.7% of patients with CRSwNP, 7.2% of patients with asthma and 14.9% of patients with severe asthma (6).

Clinical manifestations tend to appear between the third and fourth decade of life, with a moderate predominance for the female gender.

One metanalysis published in 2015 concludes that N-ERD is diagnosed in 5.5% to 12.4% of asthmatic patients, but this prevalence increases to 21% when aspirin provocation tests are performed. This would indicate that, globally, N-ERD can affect up to one in five of all asthmatic patients (7).

Reported risk factors for the disease include positive family history of N-ERD, diagnosis of CRSwNP and/or asthma, and, according to some authors, the existence of an atopic status (8).

## 3 Physiopathology

In the pathogenesis of N-ERD, it has been proposed that several environmental factors, such as viruses and allergens impact on the respiratory epithelium, inducing local release of alarmins, IL-33, IL-25 and Thymic Stromal Lymphopoietin (TSLP), that will direct T-helper cell differentiation towards a TH2 response (9, 10). Under alarmin stimulation, both type 2 innate lymphoid cells (ILC2) and mast cells amplify this TH2 response (11, 12). Particularly in N-ERD patients, PGD2 released by mast cells, combines with IL-33 in a synergistic mechanism that will further increase PGD2 concentrations. Indeed, higher expression of IL-33 and TSLP has been found in nasal polyps of patients with N-ERD than in aspirin tolerant patients (13–15).

**Figure 1:**
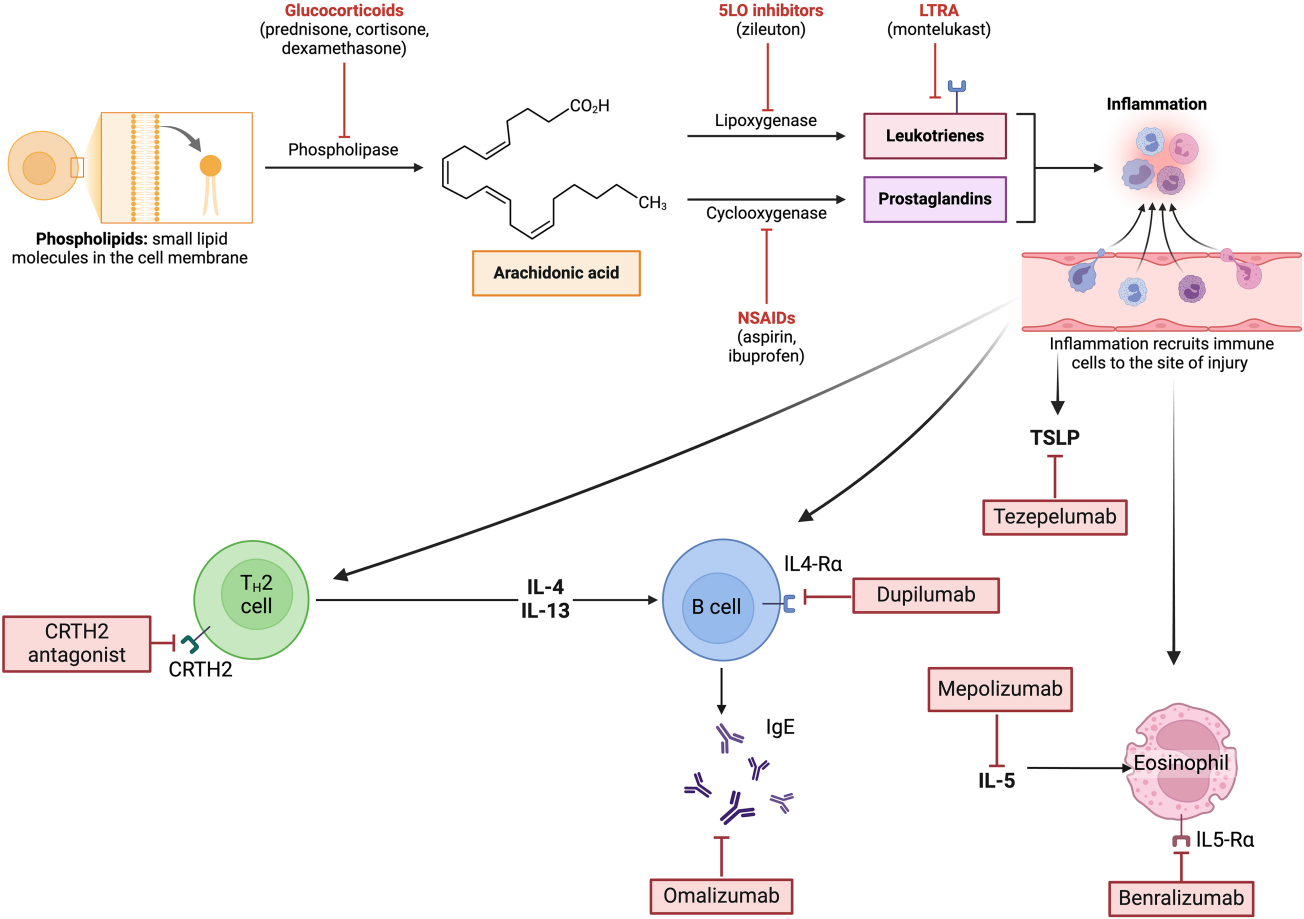
Some common medications and their role in arachidonic acid metabolism. In N-ERD, there is a preponderance of 5-LOX pathway and reduced activity of COX-1 with an overproduction of proinflammatory eicosanoids. Created with BioRender.com.

One of the main biologic characteristics of N-ERD is an imbalance of arachidonic acid metabolism, with a preponderance of 5 Lipoxygenase (5 LOX) pathway and an overall reduced activity of cyclooxygenase 1 (COX-1) (Figure 1). Further reduction of COX-1 is triggered by NSAID or aspirin intake, and arachidonic acid is then predominantly metabolized through 5-LOX, obtaining 5-Hydroperoxyeicosatetraenoic acid (5HPETE) and Leukotriene A4 (LTA4). There also exists a paradoxical synthesis of prostaglandin D2 (PGD2) because of mast cell and eosinophil activation through thromboxane receptors. Activated PGD2 receptors further stimulate TH2 cells recruitment (16, 17).

Leukotriene C4 synthase is significantly overexpressed in eosinophils and mast cells within respiratory tissue of N-ERD patients. Also, cysteinyl-Leukotriene concentrations are four times higher in this group of patients, when compared to aspirin-tolerant asthmatic patients (18, 19).

Thus, a hallmark of N-ERD physiopathology is a complex dysregulation of arachidonic acid metabolic pathways, with an overproduction of proinflammatory eicosanoids (LTC4, cysteinyl-leukotrienes and PGD2) and a deficient release and expression of anti-inflammatory mediators such as cyclooxygenase-2, PGE2, PGE2-receptor and lipoxins (20).

Chronic eosinophilic inflammation is also a paramount feature of N-ERD, with an overexpression of T2 cytokines; IL-4, IL-5, IL-13 GM-CSF, RANTES and eotaxin. Increased concentrations of IL-5 directly enhance eosinophil survival. N-ERD is also associated with increased production of IL-6, IL-13, MCP-3, CCL7, CCL5, TLR 9, and overexpression of endothelial adhesion molecules such as VCAM1, ICAM1 and E-Selectin through the inhibition of NF-KB and IKB. Some potential triggers of this inflammatory profile have been identified in N-ERD patients, were chronic viral infections and TLR3 alterations are mentioned (21–23).

There is recent evidence that patients with N-ERD present a heterogeneous set of dysregulated inflammatory cytokines, both type 2, innate lymphoid cells and IL-6 related cytokines which are elevated in the respiratory tract, oncostatin M and IL-6 overproduction are produced locally in nasal polyps and probably cause epithelial barrier disruption. Blockade of IL-4Ra, although apparently targeting T2 inflammation, also decreases innate inflammatory mediators and epithelial dysregulation (24).

Studies report a higher gene expression of GATA3, IL-4, IL-5, IL17 in patients with N-ERD, which indicates a more severe form of inflammation. The gene expression of IL-17 in the N-ERD group may suggest the coexistence of TH3 and TH2 inflammation with a predominance of Th2 inflammation (25) (Table 1).

**Table 1:** The role of main inflammatory mediators involved in N-ERD.

Inflammatory mediators	Functions and new findings
IL-33	There are several studies in which increased IL-33 has been found in the basal layer of epithelial cells in polyps from patients with N-ERD, suggesting additional expression by both fibroblasts and endothelial cells, dendritic cells and innate lymphoid cells (17, 26).
IL-25	Elevated IL-25 in the pathogenesis of eosinophilic sinonasal polyposis in Asian patients. Promotes innate type 2 inflammation (17, 26).
TSLP	Overexpressed in CRSwNP, it may contribute significantly to inflammation. It has been implicated in the pathogenesis of the disease through effects on dendritic cells and innate lymphoid cells type 2 (26).
IL-4, IL-5	Increased gene expression of GATA3, IL-4 and IL-5 in patients with N-ERD (27).
GATA 3	Increased gene expression of GATA3, IL-4 and IL-5 in patients with N-ERD, demonstrating T2 inflammation (27)
ECP, HSP70 y TRIPTASA	ECP, HSP70 and tryptase and lower levels of CC16 in nasal secretions of patients with N-ERD, suggesting increased production of mediators of eosinophil and mast cell function, and decreased production of biomarkers of respiratory epithelial function in patients with aspirin sensitivity (28).
CCL2, CXCL8, CCL5	They are found in the sinus mucosa increased in patients with N-ERD, are pleiotropic mediators and lead to large-scale inflammatory processes (29).
Mast cells	By examining CD38 and CD117 (ckit) expression, a CD38 ^high^, CD117 ^low^ (MC _t_), a CD38 ^low^ CD117 ^high^ population were identified, and a novel intermediate mast cell population expressing CD38 ^high^ CD117 ^high^ that is highly proliferative in CRSwNP and N-ERD. This suggests that local mast cell proliferation contributes to the elevated numbers of polarized mast cells observed in nasal polyps (30).
IFN-gamma	Prominent expression of IFN-gamma, which is derived from eosinophils themselves and acts in synergy with IL-3 and IL-5 to drive eosinophil maturation and these show an increased capacity not only to respond to CysLT but also to secrete cysteinyl leukotrienes (31).
IL-6	On average, however, sinonasal mucus from patients with N-ERD had significantly higher levels of IL-5, IL-6, IL-13, and IFN-*γ* than in sinonasal mucus from patients CRSwNP (27)
IL-17	Increased gene expression of IL-17 in patients with N-ERD, although significantly absent in the level of gene expression of RoRgt, may be contributing to the remodeling of nasal polyps, with accumulation of albumin and edema formation. Increased IL-17A has been shown to impact neutrophil survival in patients with CRSwNP (32).
IgE	Significant increase, due to excessive tissue overflow. IgE binds to high-affinity FceRI located on the surface of mast cells, basophils, leading to the activation of these cells that can secrete several pro-inflammatory mediators (IL-4, IL-13, histamine and eicosanoids), which can contribute to the exacerbation of symptoms (33).
Eosinophils	Higher percentage in patients with N-ERD, as well as elevated infiltration of eosinophils in nasal polyps which emphasizes Th2 inflammation, can be associated with the severity of the disease, a poor response to treatment and recurrences (34, 35).
Leukotrienes	Increased LTE4, PGD2 and decreased PGE2 and its receptor EP2 (34).

**Figure 2:**
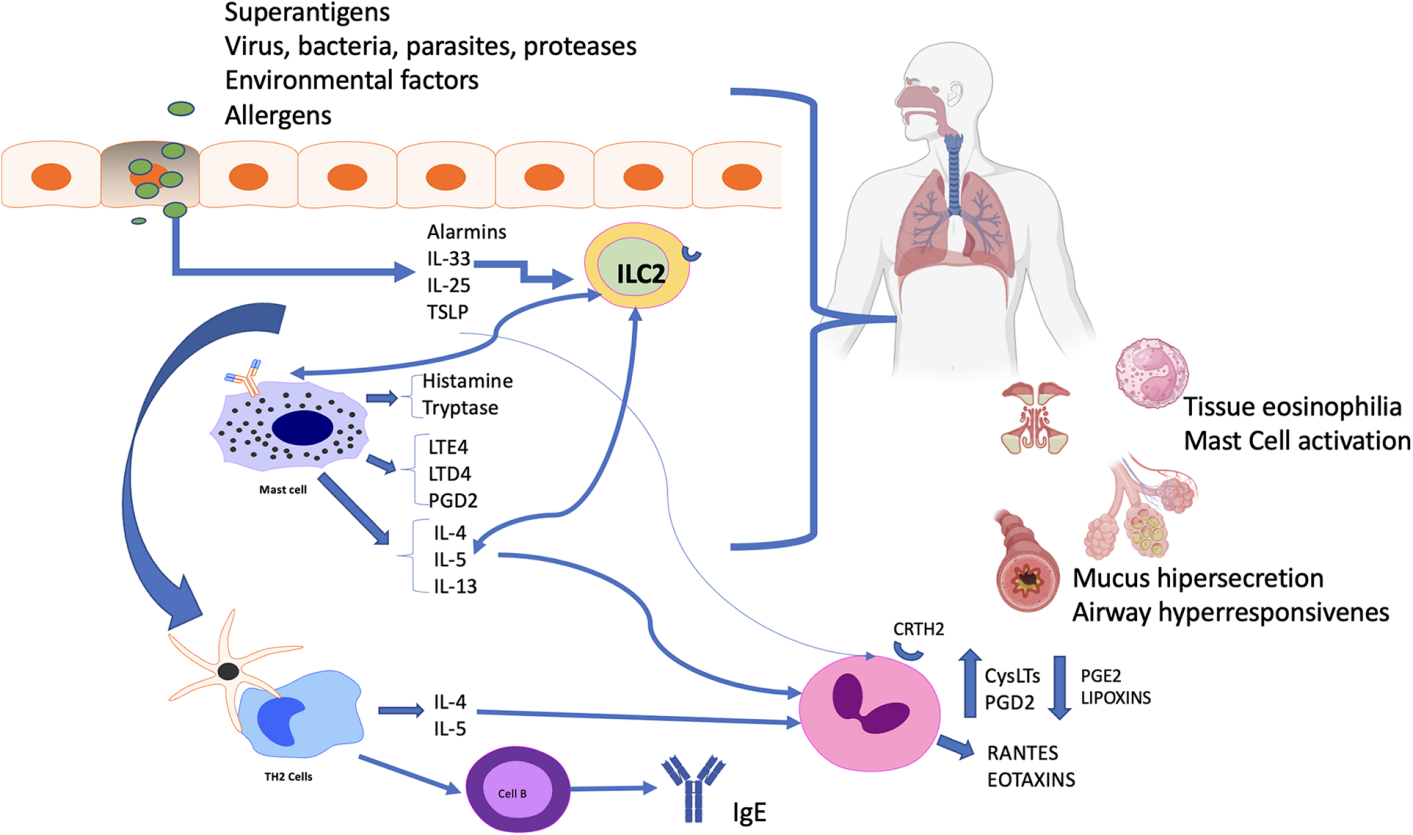
Summary of physiopathology. Allergens, superantigens, viral infection, and environmental factors are all capable of initiating epithelial injury and release of alarmins, interleukin-33, thymic stromal lymphopoietin (TSLP), and interleukin-25. These upstream cytokines have multiple effects focusing on type 2 inflammatory responses. Type 2 innate lymphoid cells (ILC2) and mast cells in N-ERD both amplify the responses, leading to eosinophilia. The combination of inflammatory mediators and cytokines, including IL-4, IL-13, and IL-31, directly affects epithelial cells, stimulating mucus overproduction, edema, and bronchoconstriction. *cysLTs*, Cysteinyl leukotrienes; *ILC2*, group 2 innate lymphoid cell; *TSLP*, Thymic Stromal Lymphopoietin; *PGD2*, prostaglandin D2; *PGE2*, Prostaglandin E2; *LTE4*, Leukotriene E4; *LTD4*, Leukotriene D4, *CRTH2*; Prostaglandin D2 receptor 2.

Another important inflammatory factor of CRSwNP in N-ERD, is the presence of tissular *Staphylococcus aureus* in around 87% of patients. This microorganism produces *Staphylococcus aureus* enterotoxin (SAE) that acts as a superantigen, triggering local allergic reactions within the ethmoid. High concentrations of specific IgE against SAE have been identified within nasal polyp tissue. These can activate TH2 cells and enhance the release of T2 cytokines and induce a strong eosinophilic response that plays an important role in the severity of both nasal polyposis and bronchial asthma (36–41) (Figure 2).

In a study made by Steinke et al, was the elevated expression of IFN-*γ* in N-ERD, consistent with recognition that this cytokine can be expressed by eosinophils. IFN-*γ* can block IgE class and it is possible that this co-expression of IFN-*γ* might contribute to the absence of allergy. Eosinophil-derived IFN-*γ* synergizes with IL-3 and IL-5 to drive maturation of eosinophils and these eosinophils display an upregulated capacity to not only respond to CysLTs but also to secrete cysteinyl leukotrienes (31).

## 4 Environmental and genetic factors

Among environmental pollutants, tobacco smoke is well recognized as one of them. In a case-control study conducted by Chang et al, a significant association (OR, 3.46; 95% CI, 2.22–5.39) was found between childhood environmental tobacco smoke (ETS) exposure and N-ERD. If a patient was exposed to ETS during both childhood and adulthood, results showed an OR of 5.09 for developing N-ERD (95% CI, 2.75–9.43) (42). Cigarette smoke stimulates COX-2 expression in airway epithelia, the development of N-ERD would be suppressed by elevated PGE _2_ levels in smokers, and smoking cessation might increase susceptibility to N-ERD. The results of a case-control study by Hayashi et al. in 2018 demonstrate that N-ERD was positively associated with smoking cessation between 1 and 4 years before disease onset (43). Tobacco smoke, secondhand smoke and occupational exposure led to increased odds for the development of N-ERD (5).

An identification of polymorphisms in LT-related genes in affected patients suggested a role for genetic variation in the development of N-ERD, this have been implicated in genes encoding enzymes synthesizing eicosanoids from arachidonic acid, related to antigen presentation and inflammation, encoding receptors for cysteinyl leukotrienes and encoding receptors that affect airway sensitivity, mucous production, lung function (44).

Epigenetics is the study of reversible molecular mechanisms that modify gene activity and expression without altering the DNA base-pair nucleotide structure, these changes are a consequence of the impact of the host environment. The influence of epigenetic changes in promoters has been shown to dramatically alter gene expression, which results in reduction in expression of PGE2 receptor 2, PGE 2 resistance, and enhanced eosinophil infiltration into respiratory tissue (45). Characteristic methylation patterns affecting 337 genes have been demonstrated, a recent study showed that there was overexpression of miR-125b in patients with eosinophilic polyposis, which plays an important role as a regulator of innate immunity through miR-125b-EIF4E binding protein 1 in the IFN pathway for mucosal eosinophilia in these patients (46).

In healthy subjects, the normal sinonasal mucosa acts as an immune mechanical barrier against pathogens. Viruses like rhinovirus and coronavirus (the most implicated in CRS) may alter host gene expression, additionally, may lead to alteration of the normal sinonasal microbiome, increased susceptibility to bacterial adherence and inhibit ciliary function leading to altered patterns of immune response and pathogenic changes in levels of cytokines/chemokines which could lead to a cascade of events causing inflammation of the upper airway (47).

## 5 Clinical presentation

N-ERD clinical symptoms usually start in the upper airway, with rhinorrhea, nasal obstruction, post-nasal drip, and smell impairment. Asthma symptoms tend to appear around two years later, usually, sinonasal polyposis is diagnosed last and around 15% of N-ERD patients present with no previous reactions to NSAIDs or aspirin and will need a provocation test to confirm the diagnosis. Hypersensitivity reactions to NSAIDs tend to appear around four years after the first upper airway symptoms (48); these include sudden nasal obstruction, watery rhinorrhea, cough, wheezing, respiratory distress and even fatal or near fatal asthmatic reactions (49).

One prospective cohort study reported by Dages et al. classified 240 patients in four groups according to the first symptom of presentation. The first group included 119 patients (50% of all patients) who presented with asthma as the first clinical manifestation. 72% of these were women, were significantly younger than patients from other groups (25 years, SE 1.3, *P* < .001) and presented with higher body mass indexes BMI [OR = 1.3 [95% confidence interval (CI): 1.06–1.7, *P* = .02]. The second group included 70 patients (29%) who presented with sinonasal polyposis as a first clinical manifestation. Of these, 70% were female and they tended to present NSAIDs intolerance at a later age (OR = 1.03, 95% CI: 1.01–1.06, *P* = .009). The third group included 39 patients (16%) who presented a NSAID-intolerance reaction as a first clinical manifestation. In this group male predominance was significant (OR = 3.3, 95% CI: 1.5–7.4, *P* = .004), and there was also significantly higher exposure to air pollution (OR = 4.4, 95% CI: 1.6–11.9, *P* = .003). The fourth group included 12 patients (5%) who presented the full triad since the beginning of the disease. 83% of these patients were female (50). This study suggests that the differences in initial clinical manifestations of N-ERD could be associated with several patient-specific risk factors that need to be studied to better categorize patient profile and enhance disease control.

A review manuscript published by Kowalski concludes that N-ERD can have very heterogeneous clinical presentations (sub-phenotypes) and possible variations in pathophysiologic mechanisms (sub-endotypes) (49). Studies from Poland and South Korea describe several clinical sub-phenotypes in terms of asthma control and severity, extension and severity of sinonasal affection, presence or absence of atopic status and of chronic urticaria. Significant differences in serum IgE, blood eosinophil count and urinary cysteinyl-leukotrienes were found between these different sub-phenotypes, thus suggesting the existence of real sub-genotypes of the disease. Understanding the clinical diversity of the disease and of its pathophysiological processes could help us better define the existing sub-phenotypes and sub-genotypes, and thus allow us to personalize treatments and improve therapeutic outcomes (49, 51).

## 6 Diagnosis and prognosis

The first step in establishing N-ERD diagnosis is an exhaustive clinical history, followed by a comprehensive physical exploration, nasal endoscopy and complementary studies such as pulmonary function testing and aspirin provocation tests.

A positive history of multiple hypersensitivity reactions, classically occurring within the first one or two hours after NSAIDs or aspirin intake, manifesting with respiratory symptoms in an adult asthmatic patient who also suffers from CRSwNP is the classical criterion for N-ERD diagnosis. Nevertheless, relying solely on these positive findings in clinical history can lead to underdiagnosis of the disease, as clinical features are not always so straightforward. Not unfrequently, an aspirin provocation test (either oral, intranasal or bronchial) is needed to confirm the diagnosis, also, one study showed that 16% of patients who reported aspirin intolerance presented negative oral provocation tests (52). Thus, an aspirin provocation test can be considered the gold standard in the diagnosis of N-ERD, as it allows to confirm or exclude ASA-hypersensitivity in patients who present with unclear history.

There are four types of aspirin provocation test: oral, intranasal, bronchial or intravenous (1).

Oral challenge is most frequently employed, as it mimics the classic exposure to medication, however, it is very time-consuming, and potential systemic reactions can prove very severe. The most positive oral provocation tests (90%–98%) include naso-ocular reaction, and 35%–90% of cases bronchial reaction, gastrointestinal, cutaneous, and laryngeal symptoms appear in 23%, 10%, and 8% of cases, respectively (35). EAACI group recommends oral challenge, starting with an initial dose of 20–40 mg of aspirin, then increasing the dose every two hours, if no reaction is seen three hours after delivering 325 mg of aspirin, the test is considered negative (7). To be considered as candidates for an oral aspirin provocation test, patients must present with a stable clinical condition and with a minimal FEV1 of 70% of the predicted value. Lower FEV1 values are a contraindication for the test (7).

Bronchial testing is also considered to be safe and fast, and a major disadvantage is that a negative result must be followed by an oral challenge test to obtain definitive diagnosis. The contraindications are basically those of spirometry itself. Oral provocation testing has been compared with bronchial provocation testing in 3 studies, both methods have the same specificity (93%), although oral provocation is more sensitive (89% vs. 77%) (53–55).

Intranasal provocation testing with lysine-aspirin is mostly recommended in patients with predominant sinonasal symptoms and is also considered a sound alternative in patients with any contraindication for oral or bronchial testing. Intranasal challenge is very safe and allows to identify a subgroup of patients who present sole intranasal reactions, but it also tends to be less sensitive than oral or bronchial challenges (7). A main limitation of this diagnostic method results from its low sensitivity, therefore, a history that is suggestive of N-ERD and a negative result in a nasal provocation still require confirmatory oral provocation. This aspirin provocation test is contraindicated in massive nasal polyposis, perforated septum, autoimmune diseases, immunodeficiency and concomitant respiratory infection (56).

Intravenous provocation testing is used exclusively in Japan, for the risk of severe adverse reaction, the IV aspirin provocation test has not been used often in clinical practice, and the safety and efficacy of this convenient method has not been assessed (57).

Characterization of the inflammatory endotype of CRSwNP is also important in N-ERD patients. The biomarkers can be obtained from peripheral blood, nasal secretions, nasal polyp tissue and even exhaled air. Peripheral blood biomarkers are easier to obtain, requiring less experience, cost and time than a nasal biopsy. However, peripheral blood biomarkers often have a poor correlation with the local sinonasal inflammatory phenomena that characterize CRSwNP (58).

Classically, CRSwNP presents as an eosinophilic disease, while CRSsNP tends to be non-eosinophilic. Classification of a rich eosinophilic milieu is established histologically through tissue microscopy. According to Kountakis et al. (59), a polyp is considered eosinophilic when 5 or more eosinophils per high-power field are found, whereas for Solear et al, a minimum of 10 eosinophils per high-power field are required (60).

Several studies have demonstrated that patients with eosinophilic CRSwNP present with higher recurrence rates, worse symptomatic and radiologic scores and higher prevalence of asthma (61, 62). Other studies correlate tissue eosinophilia and concentrations of eosinophilic cationic protein (ECP) with the severity of the disease (63).

In N-ERD, asthma is generally severe and has a T2 eosinophilic inflammatory profile and some patients present with persistent bronchospasm, rapid decrease in lung function and severe inflammation of the whole lower airway. A better understanding of the bronchial epithelial damage in N-ERD is needed to better preserve lung function on the long term (64).

Nowadays, there is no available *in vitro* test for the diagnosis of N-ERD. Urinary Leukotriene E4 (uLTE4) remains the most reliable biomarker, with higher concentrations found in N-ERD patients when compared to controls. Also, levels of uLTE4 rise significantly after aspirin or NSAIDs intake, nevertheless, this biomarker should not replace an aspirin provocation test and is not recommended for the routine diagnosis of N-ERD (29).

Imaging studies are required only when surgical treatment of CRSwNP is considered. One study demonstrated more severe ethmoidal and frontal sinuses involvement, and less maxillary sinus affection in patients with N-ERD when compared to aspirin tolerant patients with CRSwNP (65).

## 7 Treatment

There are several therapeutic options when treating N-ERD. Corticosteroids, either inhaled, intranasal or systemic, remain the cornerstone for the initial treatment; endoscopic sinus surgery is widely used in the management of CRSwNP; Aspirin desensitization remains an alternative for selected cases, and recently, monoclonal antibodies have demonstrated an important efficacy.

### 7.1 Treatment of chronic rhinosinusitis

#### 7.1.1 Medical treatment

Intranasal corticosteroids remain the gold standard of treatment of CRSwNP, and their use is linked with a decrease in inflammatory cells and proteins within the sinonasal mucosa, significantly reducing nasal symptoms such as obstruction, congestion, rhinorrhea, postnasal discharge and hyposmia. In a double blinded randomized controlled trial in N-ERD patients, Mastalerz et al. demonstrated that after a single week of intranasal fluticasone use, nasal peak inspiratory flow improved and there was a significant reduction of nasal symptomatic scores when compared to placebo (66).

The International Consensus Statement on Allergy and Rhinology recommends that nasal lavages or irrigations should be used as a first adjuvant therapeutic option for improving symptoms of CRS (66). Budesonide or other corticosteroids have been added (off label) to isotonic saline solution irrigations resulting in clinical benefit, Steinke et al., in a pilot study in subjects with eosinophilic CRS who had not responded to conventional medical therapy, were treated with budesonide nasal lavage for ≥3 months and seem to improve both olfactory function and polyp size on endoscopy and CT scan (67). However, it should be noted that this common practice is not approved by the FDA and reported results are difficult to compare because of the heterogeneity within the studies in terms of irrigation technique, type of corticosteroid, volume and dilution of the solution, rendering a metanalysis almost impossible to perform (68).

In N-ERD patients, CRSwNP and asthma tend to be quite aggressive. Systemic corticosteroids, mostly taken orally and in short courses, rapidly improve symptoms in both the upper and lower airway, but are accompanied by a wide array of endocrine, muscular, skeletal and ocular adverse effects that should limit their use as much as possible (1).

Antileukotrienes cause an important decrease in the effect and production of Cys-LTs (69). Montelukast, a direct antagonist of cys-LT1 receptor, is the most widely used agent and has been shown to improve asthma symptoms, peak expiratory flow and FEV-1 in patients with N-ERD (1). Zileuton inhibits 5-lipooxygenase, downregulating further release of Cys-LTs. Antileukotrienes help in reducing the severity and frequency of asthmatic reactions and are useful in the control of lower airway symptoms during a provocation test, nevertheless, their use during an intranasal L-aspirin provocation test can lead to false negative results (70).

#### 7.1.2 Surgical treatment

**Figure 3:**
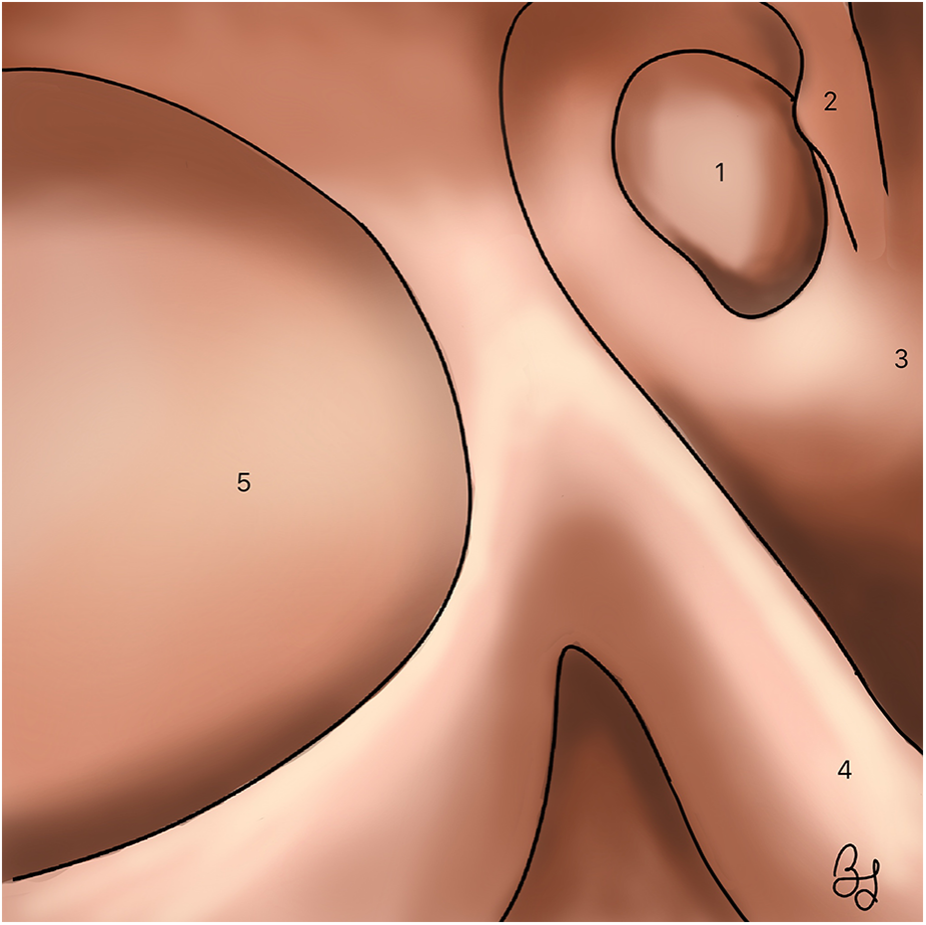
Right nasal cavity. A wide maxillary antrostomy and a sphenoidotomy are illustrated, obtaining a common cavity. (1) Sphenoid sinus. (2) Superior turbinate. (3) Septum. (4) Middle turbinate. (5) Maxillary sinus.

As previously stated, N-ERD patients tend to present with severe and diffuse forms of sinonasal polyposis that are frequently unresponsive to medical treatment and present with high recurrence rates thus requiring surgical treatment. Endoscopic sinus surgery (ESS) with maximal mucosa preservation aims to restore drainage and ventilation of the sinonasal cavities, while creating a common cavity that allows an optimal penetration of topical intranasal corticosteroids, thus enhancing inflammation control at the epithelial level (1) (Figure 3).

There is still a lot of controversy on the recommended extent of surgery for CRSwNP in N-ERD. Some authors are in favor of a “Full house ESS”, consisting of an elective complete spheno-ethmoidectomy with comprehensive dissection of the frontal recess cells (Draf II A), instead of just addressing the affected sinus cavities (71). However, one study showed that even with this “full house” technique, polyp recurrence rate is of about 58% and the need of revision surgery varies between 2 and 26.2% (72). Indeed, there is a subgroup of patients that present with highly aggressive and recurrent disease that will systematically fail to any mucosal sparing surgical treatment. Therefore, more extended and radical surgical treatments, such as nasalization, Draf III extended frontal surgery and “reboot” surgery have been proposed (73).

**Figure 4:**
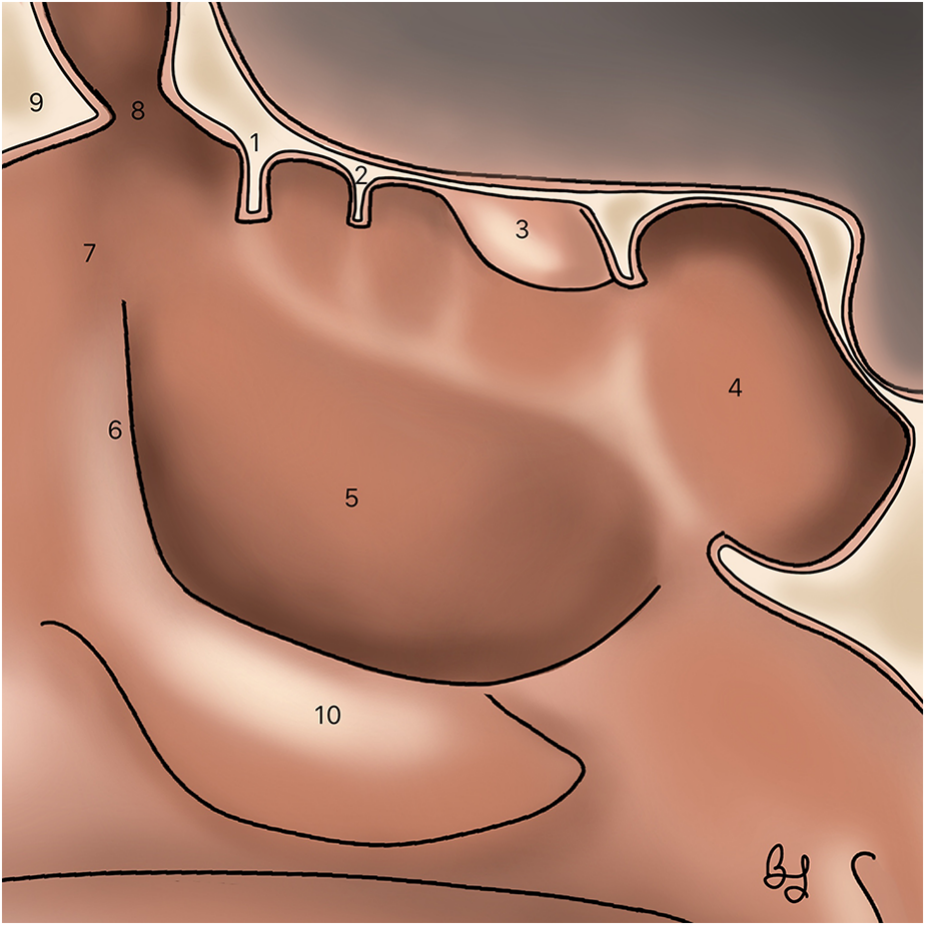
Sagittal section illustrating the classical nasalization technique. (1) Ethmoid bulla. (2) Basal lamella of the middle turbinate. (3) Superior turbinate. (4) Sphenoid sinus. (5) Maxillary sinus (6) Uncinate process. (7) Agger nasi. (8) Frontal recess. (9) Frontal Nasal Beak. (10) Inferior turbinate.

Nasalization consists of a radical ethmoidectomy with the systematic resection of all ethmoidal bony partitions and mucosa, followed by a comprehensive frontal recess dissection, a wide maxillary and sphenoid sinusotomy and middle turbinate resection (1) (Figure 4). In a prospective study on patients with diffuse sinonasal polyposis, Jankowski et al. demonstrated that nasalization had a significantly lower recurrence rate (22.7%) than classical ESS (58.3%) (74).

However, classic nasalization technique can lead to late complications such as mucoceles, nasofrontal tract stenosis or ethmoidal cavity collapse. Jiménez-Chobillon et al. proposed some modifications of the technique; the preservation of the middle turbinate and its suture fixation to the nasal septum, obtaining similar functional results with a lower complication rate (75). “Reboot” surgery was proposed by Alsharif et al. for extremely severe and recurrent polypoid disease. In this “rescue” technique, the mucosa of all the sinus cavities is eradicated as much as possible. This technique has shown further decrease of the recurrence rate at 30 months after surgery when compared with mucosal sparing techniques (76).

In a recent pilot study, Moreno-Luna et al. proposed the use of a free mucosal, harvested from the floor of the nasal fossa and adhered to the roof of the ethmoidal cavity after the full resection of the mucosa. This free mucosal flap seems to improve healing, nasal function and recurrence rates (77, 78).

**Figure 5:**
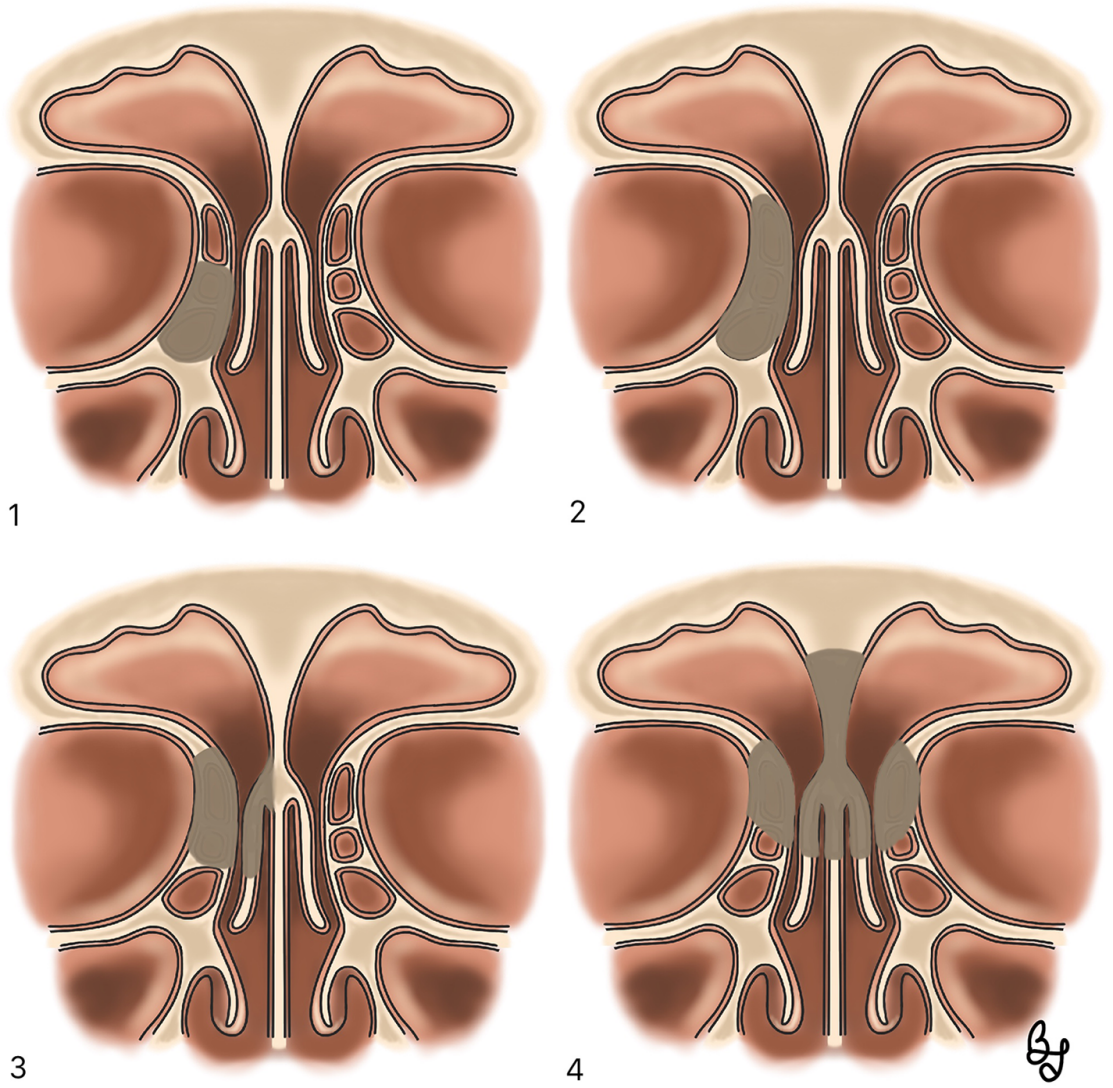
Frontal sinus surgery. (1) Draft I: anterior ethmoidectomy (2) Draft IIa: Opening of frontal sinus between lamina papyracea and middle turbinate (3) Draft IIb: Opening of frontal sinus between lamina papyracea and nasal septum (4) Draft III: removal of the frontal sinus floor and septectomy.

Diffuse polyposis and aspirin sensitivity are both independent risk factors for failure of frontal sinus surgery (79) (Figure 5). Bassiouni et al. demonstrated in a prospective study on N-ERD patients with severe polyposis that Draf III surgery (bilateral extended frontal sinusotomy with superior septectomy) was superior to Draf IIa in terms of recurrence rate (11 Vs. 55%) (80). Naidoo et al. also demonstrated that addressing frontal sinus polypoid disease with a Draf III technique enhanced the penetration of topical steroid irrigations, favoring local inflammation control and sinus ventilation (81). Noller et al. propose that Draf III surgery should be the preferred technique in cases of severe and diffuse sinonasal polyposis such as in N-ERD patients and other diseases like cystic fibrosis and primary mucociliary dyskinesia (82).

### 7.2 Aspirin desensitization

Desensitization to aspirin is another therapeutic strategy that has been used in N-ERD patients to develop drug tolerance under strict medical supervision. It has been shown to reduce polyp recurrence after ESS, increase time interval between surgeries, reduce the need for systemic corticosteroids, and improve sense of smell and quality of life (83). Several protocols have been described, with varying aspirin amounts, delivery methods, times of intake and increasing doses. Classically, desensitization is attained when a final maximal dose of 325 mg of aspirin is reached.

In a retrospective study on 32 N-ERD patients who underwent ESS and subsequent aspirin desensitization, significant improvement in SNOT-22 scores were noted and only 3 patients (9.4%) required revision surgery after 30 months of follow-up (84).

In a prospective study, Sharan et al. found that ESS could improve the effectiveness of aspirin desensitization in N-ERD, even in patients who previously failed this kind of therapy. This study suggests that ESS can bring patients to a phenotype that is more receptive to desensitization, mostly when preoperative elevated serum levels of IgE are present (85).

Despite its efficacy, treatment withdrawal rate is high because of the frequent adverse effects such as gastrointestinal disorders, bleeding and respiratory symptoms.

### 7.3 Biotherapy

**Figure 6:**
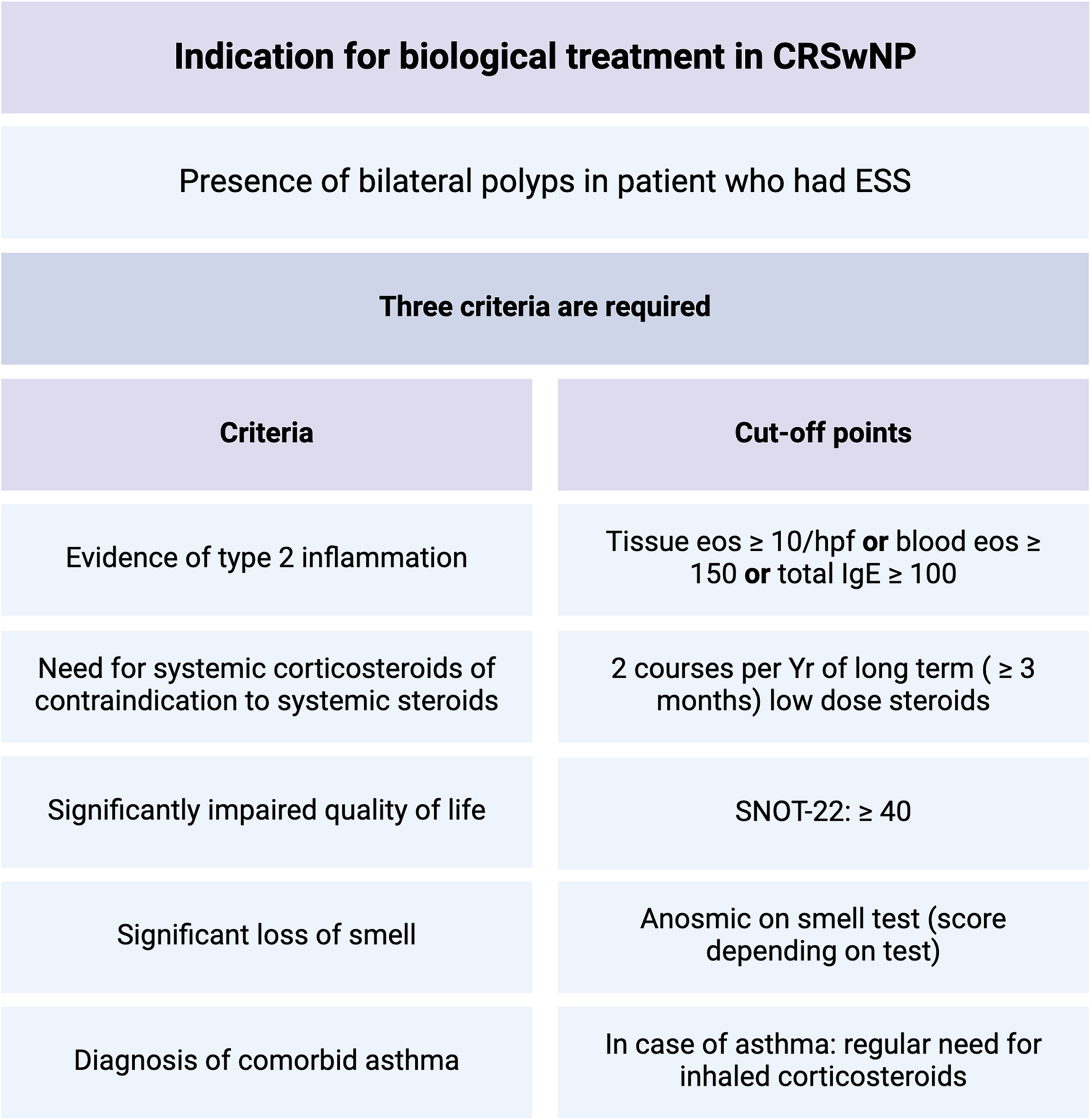
Indications for biologic agents use in CRSwNP, adapted from EPOS/EUFOREA, 2023.

Monoclonal antibodies that target type 2 inflammatory cytokines such as IL-4, IL-5 and IL-13 have gained popularity in the treatment of complex respiratory diseases. The European Position paper on Rhinosinusitis and Nasal Polyps (EPOS), in collaboration with the European Forum for Research and Education in Allergy and Airway Diseases (EUFOREA) have proposed guidelines for the proper indications and use of biologics in the treatment of CRSwNP (86) (Figure 6).

#### 7.3.1 Anti-IgE

Omalizumab is a monoclonal antibody that selectively binds to free circulating IgE and reduces the expression of IgE receptors in mast cells, basophils and dendritic cells, thus interfering with their activation. Its efficacy has been demonstrated in patients with severe asthma and other allergic diseases. In N-ERD several eosinophilic inflammatory mechanisms may be involved, thus, clinical effects of omalizumab can be quite heterogenous when compared with other forms of severe eosinophilic asthma. In a prospective cohort study, Hiyashi et al. reported a reduction of urinary LTE4 (76.2%; *P* < 0.001) and of PGD_2_M (89.0%; *P* = 0.002) after omalizumab use (87). Clinical parameters such as the number of asthmatic exacerbations, emergency department visits, systemic corticosteroids use and sinonasal and asthma symptoms scores also improved (88).

In POLYP 1 and POLYP 2 studies, omalizumab was compared to placebo in 265 patients with CRSwNP (57% with aspirin-tolerant asthma, 27% with N-ERD). 67% of patients had previous ESS. Patients who were treated with omalizumab presented a significant improvement in SNOT-22, Nasal Polyp Score and Asthma Quality of Life Questionnaire (AQLQ) (89). In a recent retrospective study by Förster-Ruhrmann et al, patients with N-ERD (confirmed by aspirin provocation test) who were treated with omalizumab presented significant clinical improvement in VAS, endoscopic staging, ACT and FEV1 during 9 months of follow up (90).

#### 7.3.2 Anti-IL-5

Nowadays, three commercially available biologics that inhibit IL-5 have been used in N-ERD patients. Mepolizumab and Reslizumab directly target IL-5, thus reducing both the production and survival of eosinophils. Benralizumab is a biologic agent that targets IL-5 receptor 5R*α* which is expressed in eosinophils, thus inducing eosinophil destruction (91).

Mepolizumab was first designed to treat severe eosinophilic asthma. However, in a randomized double blinded study by Gevaert et al, patients with CRSwNP were treated monthly with 750 mg of IV Mepolizumab and presented a significant reduction in polyp size. Five out of twenty of these patients presented N-ERD (79). In a similar study on 105 CRSwNP patients with clear indications for sinus surgery, Bachert et al. treated 54 subjects with 750 mg of IV Mepolizumab Vs. 51 placebo controls, every 4 weeks for six months. At week 25, only five patients (10%) who had received mepolizumab still needed surgery Vs. 16 patients (30%) in the placebo group (*P* = 0.006) (92). However, it should be noticed that neither of these two studies specifically assessed N-ERD patients.

In a retrospective study, Tuttle et al. studied the effects of 100 mg monthly subcutaneous injections of Mepolizumab in N-ERD patients and reported that after three or more injections, there was a significant symptomatic improvement in both the upper and lower airway (93). Indeed, prospective, double blinded clinical trials are needed to support these data on N-ERD patients.

Benralizumab has been widely used in patients with severe eosinophilic asthma, and some studies report improvement of accompanying sinonasal symptoms. However, studies that specifically assess the efficacy of this drug on N-ERD patients are needed (94, 95).

#### 7.3.3 Anti-IL4R*α*

Dupilumab is a monoclonal antibody that binds to the alpha subunit of IL-4 receptor which is also shared with IL-13, thus blocking both cytokines that are essential in T2 inflammation. In a study by Laidlaw et al. on N-ERD patients, both olfactory function (UPSIT score) and sinonasal related quality of life (SNOT-22) significantly improved after Dupilumab use (96). In two studies analyzing the cost of treatment for CRSwNP, Scangas et al. and Parasher et al. demonstrated that ESS is still a more cost-effective treatment than Dupilumab (97, 98). Thus, it is important to better define the phenotype and endotype of patients that will really benefit from a biologic treatment.

#### 7.3.4 Anti-TSLP

Tezepelumab is a recently developed monoclonal antibody that binds specifically to TSLP, blocking its action on its heterodimeric receptor. In the phase III study NAVIGATOR, Tezepelumab therapy was able to reduce asthmatic reactions and SNOT-22 score, improve lung function and normalize biomarker levels such as blood eosinophil count, FeNO, serum IgE, IL-5 and IL-13, when compared to placebo (99). In a meta-analysis published in 2023, looking at 10 randomized controlled trials, tezepelumab and dupilumab were associated with greater improvements in exacerbation rates and lung function than benralizumab or mepolizumab in subjects with eosinophilic asthma (100).

**Figure 7:**
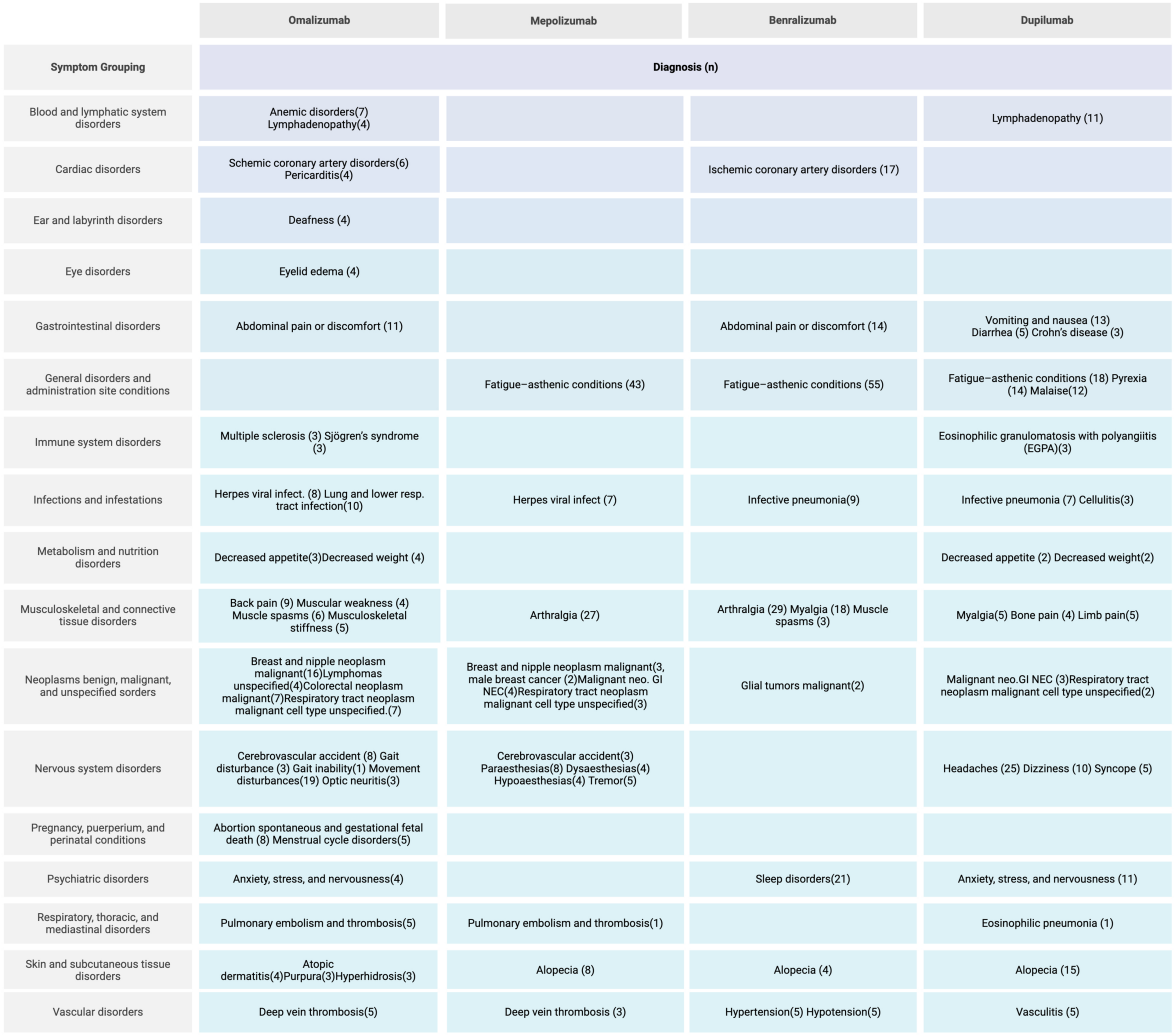
Adverse reactions of biological drug, adapted from Fernandez-del-Campo, et al.

Despite the great utility and proven benefits of monoclonal antibodies, they can have serious adverse effects that are worth knowing, which is why Fernandez-del-Campo et al. made an analysis of adverse drug reactions (ADRs) reported in the Database of the Spanish Pharmacovigilance System (101) (Figure 7).

#### 7.3.5 ASA and biotherapies

In a meta-analysis by Oykhman et al. of over 3,400 patients with CRSwNP in 29 randomized trials evaluating 9 treatment options, including ASA-D, both biologics, and ATAD, improved health-related quality of life, sinus symptoms, oral corticosteroid use, and nasal polyp size were demonstrated, with dupilumab and omalizumab demonstrating improvement in most categories evaluated (102).

In those with severe CRSwNP and eosinophilic asthma, systemic therapy is likely to significantly benefit both conditions, but the absolute benefits and cost-effectiveness are less clear. Therefore, treatment should be a decision between patients, healthcare providers, and policy makers for optimal management of CRSwNP considering benefits, costs, and adverse effects.

### 7.4 Other treatments

#### 7.4.1 Diet

Changes in nutritional habits and lifestyle can have a major impact in several chronic diseases such as diabetes or systemic hypertension. In N-ERD patients, a low salicylate diet enriched with omega-3 supplements could help in reducing the overall inflammatory status.

In a systematic review, Wood et al. were able to establish the average of daily salicylate intake in the diet of Scottish population, making it possible to define what is a high or low salicylate diet (103). However, it is difficult to apply this information to other populations because of the diversity of food and types of food processing in each country. In a multicenter prospective study, Sommer et al. evaluated the effects of a normal vs. a low salicylate diet in N-ERD patients, reporting a significant improvement in SNOT-22, Nasal Sinus Symptom Scale (NSSS) and Asthma Control Questionnaire (ACQ-7) scores in the low-salicylate diet group (104). In a prospective, randomized and two arms study, Sowerby et al. compared a high and low salicylate diet in N-ERD patients and reported that low salicylate diet group had a significant improvement in SNOT-22 scores. However, other measured parameters such as urinary salicylates and LTE4 did not reach statistical significance (105).

Due to the importance of fatty acids in the metabolic pathways of arachidonic acid, Schneider et al. studied the effects of a rich omega-3 (more than 3 g/day) and low omega-6 (less than 4 g/d) diet in N-ERD patients. They found that this diet produced a significant reduction in uLTE4 and SNOT-22 scores. However, no improvement was noted in FEV1 and FVC (106).

Alcohol intolerance has also been reported in N-ERD patients, mostly characterized by respiratory symptoms after intake. In a study on 50 patients, Cardet et al. described a 75% prevalence of alcohol induced upper respiratory symptoms such as rhinorrhea and nasal congestion in N-ERD patients. These symptoms were also present in 33% of aspirin tolerant asthmatic patients, 30% of other chronic rhinosinusitis (CRS) patients and even 14% of healthy controls. When analyzing lower airway symptoms secondary to alcohol intake, wheezing and dyspnea were present in up to 51% of N-ERD patients, and in 20% of aspirin tolerant asthmatic patients. No CRS patient or healthy controls presented lower airway affection after drinking alcohol (107).

Mechanisms of alcohol induced airway symptoms are not fully understood. Candelo et al. describe that phenols such as resveratrol found in beer and red wine can induce COX-1 inhibition, thus leading to the same pharmacologic effects than aspirin or NSAIDs (108). In a prospective cohort study, Glicksman et al. found that aspirin desensitization can improve tolerance to alcohol intake (109). Also, Mustafa et al. describe that 70% of N-ERD patients develop alcohol tolerance after initiating Dupilumab therapy (110).

#### 7.4.2 New treatments

The cellular effector CRTH2 + has been reported to be involved in PGD2 mediated inflammation, in asthma, expression of the prostaglandin D 2 receptor 2 (DP 2 receptor) is increased in the bronchial submucosa and DP 2 receptor stimulation by prostaglandin D 2 mediates the activation and migration of Th2 cells, ILC2, basophils, and eosinophils, and stimulates type 2 cytokine release from these cells, as well as the migration of airway smooth muscle cells (111), the findings implicate the prostaglandin D2—DP 2 axis in the pathogenesis of asthma. On the other side, DP 2 expression is algo associated with CRS, including nasal polyps formation (112), so the pathophysiology of CRSwNP and asthma are similar in terms of the involvement of the DP 2 receptor pathway. A newly designed agents such as feviviprant are targeted against this molecule. In LUSTER-1 and LUSTER-2 studies, feviviprant was compared to placebo in subjects with severe asthma with modest reductions in exacerbations rates were observed in both studies with the 450 mg dose of fevipiprant (113). In THUNDER study this monoclonal antibody was compared to placebo in subjects with CRSwNP and concomitant asthma measured by improvement in nasal polyp score (primary end point), nasal congestion score, SNOT-22, and UPSIT score with no clinical effect and the main limitation of this study were that only 1 patient had a diagnosis of N-ERD (114).

Feviviprant may have a role in the treatment of N-ERD, where it is thought that the role of DP2 receptor pathway be more important supported by the fact that N-ERD subjects have elevated levels of urinary PGD2 metabolites compared to aspirin-tolerant asthma subjects, furthermore, suppression of PGD2 in N-ERD subjects by high-dose aspirin therapy is understood to be one of the mechanisms of benefit of high-dose aspirin therapy in N-ERD (115, 116).

Finally, the inflammatory role of platelets through the activation of thromboxane receptors has been investigated in N-ERD. Ifetroban, a thromboxane receptor antagonist would attenuate the severity of sinonasal, and respiratory symptoms induced during aspirin challenges in patients with N-ERD by blocking platelet activation, reducing cysLT generation, and reducing aspirin-induced bronchoconstriction. A trial conducted in 2024, ifetroban was compared to placebo in subjects with N-ERD where worsening of aspirin-induced reactions was observed with possible ablation of the low-level protective PGE2 effect (117, 118).

### 7.5 Effectiveness and cost-effectiveness

Worldwide, many health systems, especially public ones, are under high demand and often have limited budgets to provide the necessary services. As already mentioned, there are different treatment modalities for N-ERD, of which monoclonal antibodies are currently the most expensive therapeutic measures, so it is important to assess their cost-effectiveness and efficacy against other forms of treatment to promote the sustainability of health care.

Some cost-effectiveness studies on CRSwNP suggest that FEES**,** including revision surgery, is more cost-effective than dupilumab, however these analyses do not consider other factors such as the number of prior surgeries, nasal polyp scores, initial SNOT-22 scores, and the proportion of patients with concomitant asthma, so the true cost-benefit of monoclonal antibodies in N-ERD is still unknown (Table 2).

**Table 2:** Summary of studies on effectiveness and cost-effectiveness of different treatment options.

Author, year	Type of study	Population	Outcome	Findings
Yong et al. 2023 (119)	Cost-effectiveness analysis	Subjects with CRSwNP which was refractory to initial FESS and appropriate medical management. Dupilumab, omalizumab and mepolizumab were compared	Incremental cost per quality-adjusted life year calculated from SNOT-22, illustrated by the incremental cost- effectiveness ratio	Omalizumab is the most cost-effective biologic in terms of cost and efficacy in CRSwNP
Yong et al. 2021 (119)	Cost-effectiveness analysis	Subjects diagnosed with CRSwNP in the setting of N-ERD who previously failed appropriate medical management, who may or may not have had previous nasal polyp surgeries.	Incremental cost per quality-adjusted life year calculated from SNOT-22, illustrated by the incremental cost- effectiveness ratio	Dupilumab is a cost-effective treatment as a salvage option for CRSwNP when compared with ASA desensitization after FESS
Scangas et al. 2021 (97)	Cost-effectiveness analysis	Subjects with CRSwNP through two treatment strategies: dupilumab versus FESS plus postoperative medical therapy	Incremental cost per quality-adjusted life year calculated from SNOT-22, illustrated by the incremental cost- effectiveness ratio	Both primary and revision FESS to be a more cost-effective treatment option compared to dupilumab for CRSwNP patients
Parasher et al. 2022 (98)	Cost-effectiveness analysis	Subjects diagnosed with CRSwNP refractory to medical therapy through two treatment strategies: dupilumab versus FESS	Incremental cost per quality-adjusted life year calculated from SNOT-22, illustrated by the incremental cost- effectiveness ratio	Dupilumab and FESS may demonstrate similar clinical effectiveness, FESS remains the most cost-effective treatment option for patients with CRSwNP refractory to medical therapy.
Ali et al. 2024 (120)	Cost-effectiveness analysis	Subjects with severe uncontrolled type 2 asthma through five treatment strategies: dupilumab, omalizumab, mepolizumab and benralizumab	Incremental cost per quality-adjusted life year calculated from exacerbation management, and follow-up costs, illustrated by the incremental cost- effectiveness ratio	Dupilumab 200 mg was strongly dominant versus omalizumab 450 mg and 600 mg, mepolizumab 100 mg, and benralizumab 30 mg; however, cost-effectiveness was not demonstrated versus omalizumab 300 mg
Staufenberg et al. 2024 (121)	Retrospective multicenter study	Subjects with N-ERD who has been prescribed dupilumab, omalizumab, and mepolizumab	Endoscopic polyp score, SNOT-22 questionnaire score, visual analogue scoring of total symptoms/severity of disease, and sense of smell	All outcomes improved significantly after 4 and 12 months of add therapy (*p* = 0.0001) with all biologics
Wangberg et al. 2021 (122)	Retrospective study	Subjects with N-ERD who has been prescribed omalizumab, mepolizumab, reslizumab, benralizumab or dupilumab	SNOT-22 scores, systemic corticosteroid, antibiotic prescriptions, and emergency room visits related to N-ERD pre and 12 months after biologics	Anti-IL-4R therapy led improvement in SNOT-22 (*p* = 0.0002), corticosteroid bursts (*p* ≤ 0.0001), median number of antibiotic courses for respiratory disease (*p* = 0.0468) when compared with anti-IL-5/IL-5R and anti-IgE biologic therapies
Laidlaw et al. 2019 (96)	Randomized Controlled Trial	Subjects with N-ERD (8 in dupilumab-treated group and 11 in the placebo group)	Lund-Mackay score, FEV1, SNOT-22 and UPSIT.	Dupilumab significantly improved CRSwNP disease with comorbid N-ERD outcomes
Tuttle et al. 2018 (93)	Retrospective study	Subjects with N-ERD who received 3 or more mepolizumab doses	Demographic characteristics, medications, AEC, SNOT-22, ACT, and FEV1 before initiation and after 3 or more doses of mepolizumab.	AEC, SNOT-22 decreased significantly (*p* < .01, *p* < .01). Treatment with mepolizumab significantly increased overall ACT scores increased (*p* = 0.002) and there was an improvement in FEV1% predicted (*p* = 0.16)
Jean et al. 2019 (123)	Retrospective study	Subjects with N-ERD who has been prescribed omalizumab	Number of oral steroid courses, SABA, canisters used, emergency department visits, antibiotics for sinusitis or pneumonia, pulmonary function tests compared between 6 and 12 months before starting and during the last 6 and 12 months on omalizumab	Reduction in the number of steroid courses (*p* = 0.0014) and number of SABA canisters used (*p* = 0.0005) during their last 12 months while on omalizumab.
Cooper et al. 2019 (124)	Prospective cohort study	N-ERD subjects underwent ASA desensitization	SNOT-22, ACQ responses, acoustic rhinometry, peak flow readings, and endoscopic scoring of nasal polyps were recorded prior to desensitization and after 6 months of maintenance therapy.	Improvement after 6 months of maintenance therapy to a median SNOT-22 score of 18.5 ± 17.3 (*p* = 0.025)
Mortazavi et al. 2017 (125)	Randomized double-blind placebo control clinical trial	N-ERD subjects. Active group went through aspirin desensitization and the control group received placebo	Six months after, asthma attacks, recurrence of nasal polyposis, FEV1, SNOT-22, Lund-Mackay score, levels of IL-4, IL-5	SNOT-22, medication requirements, symptom score and IL-5 was significantly lower in the active group after six months (*p* = 0.001, 0.005, 0.017, 0.019 respectively). FEV1 was higher in the active group (*p* = 0.032)
Lee et al. 2019 (126)	Retrospective study	N-ERD subjects who underwent FESS with at least 6 months of pre- and postoperative clinical data. Zileuton cohort chosen on intention-to-treat after FESS	RSDI scores, antibiotics use, corticosteroid use, otolaryngology visits, and time to revision surgery	Zileuton therapy for N-ERD patients shows no statistical benefit for rhinologic quality-of-life symptoms
Sommer et al. 2016 (104)	Prospective crossover single-blind multicenter study	N-ERD subjects were randomized to start with either 6 weeks of a regular diet or 6 weeks of a low-salicylate diet and then crossed-over for a total study duration of 12 weeks.	SNOT-22, NSSS, ACQ, POSE and LKES	Subjects had improvement in SNOT-22: 15 [95% confidence interval (CI), 10 to 23.25], *p* < 0.001; NSSS: 3 (95% CI, 1.75 to 4), *p* < 0.001; ACQ-7: 4.5 (95% CI, 1.5 to 8.5), *p* < 0.001; POSE 6 (95% CI, 2.5 to 10), *p* < 0.001; and LKES: 2.5 (95% CI, 1.5 to 4), *p* < 0.001).
Kęszycka et al. 2021 (127)	Prospective, nonrandomized, baseline-controlled intervention study	Subjects with N-SAIDs hypersensitivity. Diet adherence and salicylate intake were measured by a 3-day food record	ACT, SNOT-22 and FIIQ	ACT score increase (*p* < 0.002), the SNOT-22 and FIIQ score decreased after a dietary intervention (*p* < 0.0002 and < 0.0002).
Adelman, et al. 2016 (128)	Systematic review	Studies with both preoperative and postoperative data for patients with N-ERD who underwent sinus surgery	Sinonasal and asthma symptom scores	Improvement in sinonasal and asthma symptom severity and frequency, radiographic and endoscopy scores, and quality of life after surgery.

SNOT-22, sino-nasal outcome test—22; SABA, short-acting-agonist; ACQ, asthma control questionnaire; FEV1, forced expiratory volume in the second one; FEES, functional endoscopic sinus surgery; RSDI, rhinosinusitis disability index; NSSS, nasal sinus symptom scale; POSE, perioperative sinus evaluation; LKES, Lund-Kennedy endoscopy score; ACT, asthma control test; FIIQ, four-item itch questionnaire; AEC, absolute eosinophil count; UPSIT, University of Pennsylvania smell identification test.

## 8 Conclusions

N-ERD is a very complex and heterogenous disease that requires a multidisciplinary approach, involving pneumologists, allergists, ENT-surgeons among other medical specialties to attain optimal results in terms of control and quality of life. When comprehensive medical treatment fails, surgery is indicated, and although conservative surgeries are intended as they present fewer complications, now a days, ethmoidal radical surgical techniques are recommended for the control of CRSwNP in N-ERD.

In recent years, a better understanding of the mechanisms involved in T2 inflammation has allowed the development of several more targeted therapies, such as monoclonal antibodies targeting T2 inflammation cytokines, which offer a new paradigm in treatment and still need to be investigated to obtain their true long-term efficacy. The genetics and epigenetics of N-ERD need to continue to be studied to obtain more personalized and targeted therapies, therefore, more effective in the future.
